# Determinants of combination GM-CSF immunotherapy and oncolytic virotherapy success identified through *in silico* treatment personalization

**DOI:** 10.1371/journal.pcbi.1007495

**Published:** 2019-11-27

**Authors:** Tyler Cassidy, Morgan Craig

**Affiliations:** 1 Department of Mathematics and Statistics, McGill University, Montreal, Quebec, Canada; 2 Département de mathématiques et de statistique, Université de Montréal, Montreal, Quebec, Canada; 3 Department of Physiology, McGill University, Montreal, Quebec, Canada; Harvard Medical School, UNITED STATES

## Abstract

Oncolytic virotherapies, including the modified herpes simplex virus talimogene laherparepvec (T-VEC), have shown great promise as potent instigators of anti-tumour immune effects. The OPTiM trial, in particular, demonstrated the superior anti-cancer effects of T-VEC as compared to systemic immunotherapy treatment using exogenous administration of granulocyte-macrophage colony-stimulating factor (GM-CSF). Theoretically, a combined approach leveraging exogenous cytokine immunotherapy and oncolytic virotherapy would elicit an even greater immune response and improve patient outcomes. However, regimen scheduling of combination immunostimulation and T-VEC therapy has yet to be established. Here, we calibrate a computational biology model of sensitive and resistant tumour cells and immune interactions for implementation into an *in silico* clinical trial to test and individualize combination immuno- and virotherapy. By personalizing and optimizing combination oncolytic virotherapy and immunostimulatory therapy, we show improved simulated patient outcomes for individuals with late-stage melanoma. More crucially, through evaluation of individualized regimens, we identified determinants of combination GM-CSF and T-VEC therapy that can be translated into clinically-actionable dosing strategies without further personalization. Our results serve as a proof-of-concept for interdisciplinary approaches to determining combination therapy, and suggest promising avenues of investigation towards tailored combination immunotherapy/oncolytic virotherapy.

## Author summary

The advent of biological therapies for anti-cancer treatment has had a significant impact on patient outcomes. Targeted xenobiotics, including oncolytic viruses, in combination with existing, more general, immunotherapies like exogenous cytokines show great promise for continuing to improve cancer care. However, determining optimal combination regimens can be difficult, given that testing proposed schedules would require large cohorts of patients enrolled in clinical trials. Fortunately, computational biology can help to address treatment scheduling while simultaneously helping to unravel the mechanisms driving therapeutic responses. In this work, we integrate a mathematical model of GM-CSF and talimogene laherparepvec (T-VEC) oncolytic virotherapy into a virtual clinical trial to optimize their administration in combination. Using this platform, we inferred a clinically-actionable combination schedule for patients with late-stage melanoma that significantly improved virtual patient outcome when compared to GM-CSF and T-VEC monotherapies, and a standard combination strategy. Our results outline a rational approach to therapy optimization with meaningful consequences for how we effectively design and implement clinical trials to maximize their success, and how we treat melanoma with combined immuno- and virotherapy.

## Introduction

Modern cancer treatments increasingly incorporate a broad class of biological therapies known as immunotherapies to activate the immune system against cancer cells in a generalized or targeted way [1, 2]. These therapies seek to exploit existing tumour-immune interactions to more effectively recognize and destroy tumour cells with the goal of minimizing off-target and detrimental side effects. Current and investigational immunotherapies include immune-checkpoint inhibitors, monoclonal antibodies, CAR-T cells, and the exogenous administration of cytokines. One such cytokine, granulocyte-macrophage colony-stimulating factor (GM-CSF), is a white blood cell growth factor responsible for stimulating granulocyte production, and orchestrating innate inflammatory responses. GM-CSF has been used to increase the efficacy of monoclonal antibodies, and has also been administered during B-cell lymphoma treatment to activate certain immune cell subsets [2].

Another older idea, recently adopted in clinical applications, is to use oncolytic viruses to destroy tumour cells [3, 4] and activate an immune response. Oncolytic viruses are genetically engineered to preferentially attack and infect cancerous cells [5, 6], forcing infected cells to undergo lysis and release tumour specific antigens that signal the immune system to mount an anti-tumour response [7, 8]. This double effect against tumour cells has encouraged the study of oncolytic viruses as a treatment against a variety of malignant solid tumours. In 2015, the modified herpes simplex virus talimogene laherparepvec (T-VEC) was the first oncolytic virus to be approved by the Food and Drug Administration in the United States for use in patients with non-resectable melanoma [9–11]. T-VEC is specifically engineered to enhance expression of GM-CSF after viral infection of tumour cells [9]. However, despite much promise, the efficacy of oncolytic virus monotherapy has been limited [8, 12, 13]. As it is reasonable to expect that immunotherapy and virotherapy could act synergistically to instigate an immune response against tumour cells [14–16], recent efforts have focused on determining the anticipated benefit to their use in combination with a variety of immunotherapies [17, 18]. To that end, GM-CSF has been considered as an immune stimulant during oncolytic virotherapy [2].

Combination therapy can carry a high therapy burden and may increase overall toxicity [13, 18]. Unfortunately, running clinical trials for all possible (dose, time)-pairs of a proposed combined treatment to determine efficient and safe scheduling is both time and cost prohibitive. Consequently, regimen scheduling of combination immuno-/oncolytic virotherapy remains an open problem. There is an established history of applications of modelling-based, computational biology approaches to the *in silico* determination of potential therapeutic schedules that concretely improve patient outcomes [19–22]. In a closely related recent paper, an *in silico* clinical trial approach to anti-CTLA-4 and anti-PD-L1 scheduling in breast cancer demonstrated how systems pharmacology can be leveraged for therapy individualization, subsequently increasing our understanding of the optimization of combination immunotherapy treatment [23]. Similarly, by employing a straight-forward evolutionary game theory model to determine adaptive treatment schedules, Zhang et al. [24] reported significant improvements to prostate specific antigen in comparison to the standard-of-care in an on-going phase I clinical trial. These and other [25] successes motivate the continued application of interdisciplinary approaches in personalized oncology. Perhaps the most significant impact made by quantitative methodologies is the identification and translation of the underlying determinants of treatment success into actionable therapeutic strategies [26, 27].

To that end, here we detail the rationalization of combination immuno-/virotherapy scheduling for patients with late-stage melanoma by implementing an *in silico* clinical trial. By integrating our previous computational biology model of sensitive and resistant tumour cells and their interactions with the immune system into our virtual trial platform, we generated identical virtual patient cohorts to determine optimal, individualized treatment regimens for combined GM-CSF immunotherapy and T-VEC. We used the results of the personalization to infer a logical and clinically-actionable dosing scheme that significantly improved overall survival and progression free survival while substantially reducing drug burden. Crucially, we identified key mechanisms that determine therapy success, which allowed us to define a successful regimen in a new cohort of virtual patients. Our results highlight the potential and potency of rational regimen prediction using a computational biology approach, and serve as a proof-of-concept for future quantitative studies in oncology.

## Methods

### Computational biology model

**Fig 1:**
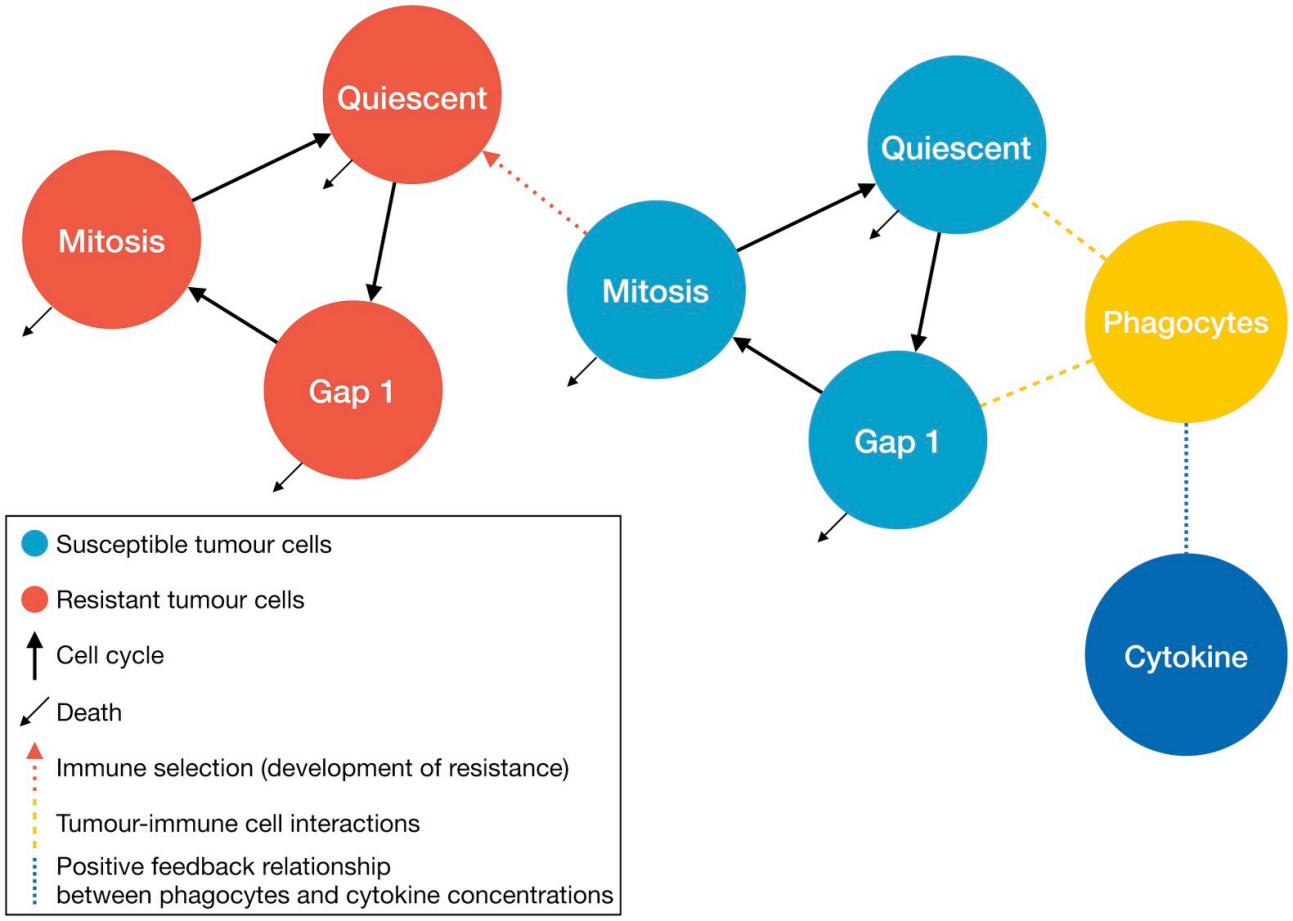
Pictorial representation of the tumour growth model. Quiescent cells activate to begin division by transiting into the *G*_1_ phase of the cell-cycle. Cells exit *G*_1_ to enter the active phase and complete division. Most susceptible cells in the active phase re-enter quiescence after mitosis, however certain dividing cells may mutate into an immuno-resistant lineage (red dotted arrow). Immune interactions are driven by phagocytes who come into contact with quiescent and *G*_1_ phase susceptible cells (dashed yellow lines). Tumour-immune interactions increase pro-inflammatory cytokine concentrations to recruit additional phagocytes to the tumour site (blue dotted line). Cells and cytokine are denoted by circles, processes by squares, and rates by arrows.

To establish the synergistic interactions elicited between immunotherapy (exogenous GM-CSF) and oncolytic virotherapy, we adapted our previous mathematical model [28] describing the instantaneous change in tumour size, phagocyte numbers and cytokine concentrations over time. Here, GM-CSF acts as a cipher for a generic immunostimulatory cytokine, selected to investigate the role of immunostimulation on therapy success, given its specific role in T-VEC administration (namely that T-VEC is modified to enhance GM-CSF secretion). The model (Eq (1)) tracks both immuno-susceptible and immuno-resistant tumour cell populations as they progress through the cell cycle. Quiescent immuno-susceptible tumour cells (denoted by *Q*(*t*)) can be cleared through either random death or immune pressure at rates *d*_1_ and *φ*_*Q*_ respectively, or transit into the *G*_1_ phase to begin reproduction at rate *a*_1_. Cells in *G*_1_, denoted by *G*_1_(*t*), are also subject to random death and immune clearance at rates *d*_2_ and *φ*_*G*_ respectively before beginning the mitotic process at rate *a*_2_. Cells in *G*_1_ or the mitotic portion of the cell cycle are susceptible to infection by the oncolytic virus at rate *η*. After completing division, mitotic cells return to quiescence. While we do not explicitly consider mutations that may increase immune recruitment, mitotic cells may mutate at rate *μ* into an immuno-resistant cell type with a low probability. This immuno-resistant lineage maintains the same cell cycle behaviour of non-resistant tumour cells, but evades immune pressure and is therefore not subject to any immune interactions. We do not distinguish between different types of immune cells in the tumour microenvironment, but rather model all phagocytes as a single population denoted by *P*(*t*). These immune cells interact with the susceptible tumour cell population and produce a pro-inflammatory cytokine (e.g. interleukin-12, tumour necrosis factor, interferon gamma, GM-CSF etc.) to recruit other phagocytes to the tumour site. Here, we denote the pro-inflammatory cytokine concentration by *C*(*t*). The mathematical model includes a distributed delay term that represents the heterogeneous cell cycle duration of cancerous cells, and is derived in Cassidy and Humphries [28]. The various interactions described above are schematized in Fig 1.

$$\begin{array}{lll} \frac{\text{d}}{\text{dt}}Q(t) & = & 2(1-\mu )\int_{-\infty }^{t}[\text{exp}\left(-\int_{\sigma }^{t}{\hat{d}}_{K}+\eta (U(x))+\psi_{G}(U(x))\text{d}x\right)\\ & & \times a_{2}G_{1}(\sigma )K(t-\sigma )]\;\text{d}\sigma -\left[a_{1}+d_{1}+\psi_{Q}(U(t))\right]Q(t)\\ \frac{\text{d}}{\text{dt}}G_{1}(t) & = & a_{1}Q(t)-\left[a_{2}+d_{2}+\eta (V(t))+\psi_{G}(U(t))\right]G_{1}(t)\\ \frac{\text{d}}{\text{dt}}Q_{R}(t) & = & 2\mu \int_{-\infty }^{t}[\text{exp}\left(-\int_{\sigma }^{t}{\hat{d}}_{K}+\eta (U(x))+\psi_{G}(U(x))\text{d}x\right)\\ & & \times a_{2}G_{1}(\sigma )K(t-\sigma )]\;\text{d}\sigma -\left[a_{1}+d_{1}\right]Q_{R}(t)\\ & & +2\int_{-\infty }^{t}\text{exp}\left[-\int_{\sigma }^{t}{\hat{d}}_{K}+\eta (U(x))\text{d}x\right]a_{2}G_{1,R}(\sigma )K(t-\sigma )\text{d}\sigma \\ \frac{\text{d}}{\text{dt}}G_{1,R}(t) & = & a_{1}Q_{R}(t)-\left[a_{2}+d_{2}+\eta (V(t))\right]G_{1,R}(t)\\ \frac{\text{d}}{\text{dt}}I(t) & = & \eta (V(t))\left[G_{1}(t)+G_{1,R}(t)+N(t)\right]-\delta I(t)\\ \frac{\text{d}}{\text{dt}}V(t) & = & {\text{Dose}}_{V}(t)-\eta (V(t))\left[G_{1}(t)+G_{1,R}(t)+N(t)\right]+\alpha [\delta I(t)]-\omega V(t)\\ \frac{\text{d}}{\text{dt}}C(t) & = & {\text{Dose}}_{C}(t)+C_{prod}(U(t))-k_{elim}C(t)\\ \frac{\text{d}}{\text{dt}}P(t) & = & \varphi (C(t))-\gamma_{p}P(t). \end{array}\}$$ (1)

Model predictions are obtained by reducing the distributed DDE to an equivalent finite dimensional system as previously described [28, 29]. Full details are provided in the Supplementary Information file S1 Text.

### Generation of *in silico* individuals and patient cohorts

To calibrate the model to available data, we adopted a sequential fitting procedure to parameterization. We used time series data from a number of experimental settings to estimate the different model parameter values. We began by determining the parameters of the delay kernel *K*(*t* − *σ*) using data from a cervical cancer cell line [30], before fitting the remaining parameters in a sequential manner. First, data from tumour growth in immuno-compromised mice was used to fit the tumour growth parameters [31]. Next, we fit the viral kinetic parameters using a combination of *in vitro* data from Toda et al. and Randazzo et al. [32, 33]. Finally, we used data from GM-CSF concentrations following administration of a T-VEC precursor in mice to fit the parameters for the cytokine compartment. In each case, we reduced the mathematical model to replicate the experimental set up, and minimized the least-squares error between simulations and experimental data (S1 Text).

To reflect the inter-individual variability and heterogenous nature of patient cohorts, we individualized the model by generating a unique set of parameters to represent a single patient. To create individuals in the *in silico* clinical trial, we sampled the model’s parameters from a generated normal distribution with mean $\hat{\mu }$ determined in the sequential fitting procedure. We defined **p** to be the vector of fitted parameter values and parameterized the normal distributions so that 99.7% of patients fall within $\left[\hat{\mu }-3\sigma ,\hat{\mu }+3\sigma \right]=\left[0.5\text{p},1.5\text{p}\right]$. If empirical information about a parameter’s distribution was available, this measurement was used in lieu of the previously described procedure. Virtual individuals are then created by sampling each model parameter from this distribution. We confirmed that using this methodology created virtual patients with parameter values following an approximately normal distribution about the mean empirical or fitted value (S4 Fig). The distribution of parameters approximates the empirical distribution used to define the virtual population, indicating that this virtual patient generation procedure produces a representative sample of the possible virtual population (and not multiples of the same individual).

To further protect against the creation of nonrealistic virtual patients, we imposed selection and inclusion criteria on each generated individual by verifying that each virtual patient responds in a physiologically-realistic way without and with treatment [19]. Specifically, we compared the predicted response of each virtual patient to currently approved oncolytic virotherapy for stage IIIb or IV non-surgically resectable melanoma [8, 9]. Moreover, we assessed whether the predicted tumour doubling time of each individual corresponded to clinically relevant tumour doubling times [34], and used this comparison as the sole inclusion criterion for subsequent enrolment *in silico* clinical trial simulations. To ensure that we were sampling from the entirety of the physiologically realistic portion of parameter space [19], we performed a local sensitivity analysis to determine the impact of parameter variation on model output (S1 Text).

**Fig 2:**
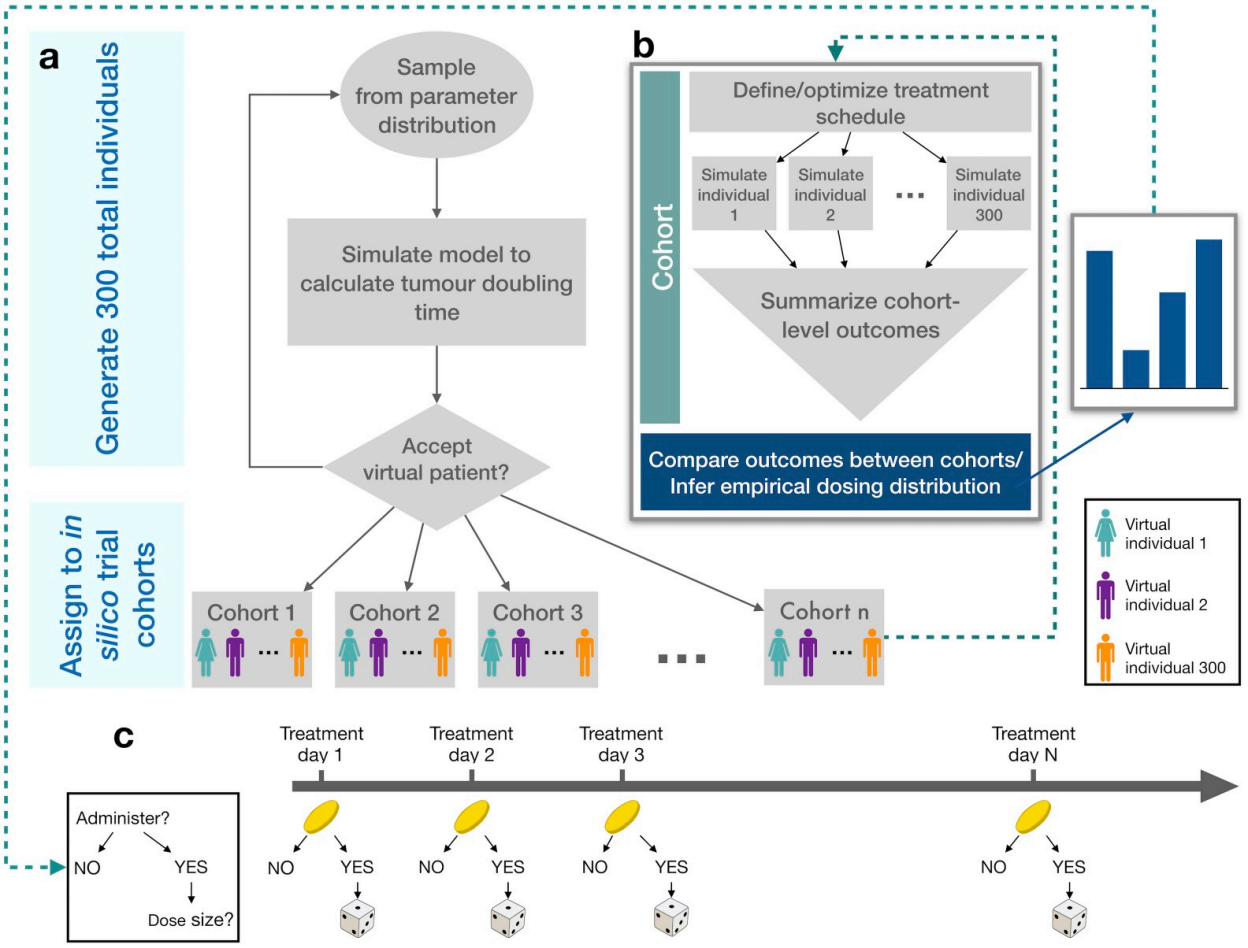
Summary of *in silico* clinical trial platform. a) Individual *in silico* patient parameter values are sampled from a normal distribution of values based on an average parameterization. The model is then simulated for each individual and predictions are tested for physiological relevance. If realistic, the virtual individual is cloned n times and each clone is assigned to n separate cohorts. b) Each cohort undergoes a different treatment protocol, from which summary statistics are collated and compared between other cohorts. Cohorts may also undergo therapy optimization (see Methods) from which an empirical distribution for the probability of administering a given dose is inferred. c) The probabilistic treatment protocol is based on probability distributions inferred from the individualization based on procedures in a and b.

We accepted a total of 300 virtual patients generated by the parameter sampling and selection processes outlined above. Each virtual patient was then reproduced into *n* identical clones, and each resulting clone was subsequently assigned to one of *n* separate cohorts (for example, a treatment free control group, a mono-immunotherapy group, and an oncolytic virotherapy group, for a total of *n* = 3 cohorts). In this way, the total number of participants is 300 times the total number of simulated investigational arms, or 300*n*. For each of the 300*n* virtual patients, we simulated the mathematical model to determine disease progression during treatment (see S1 Text for simulation details). The *in silico* trial generation and simulation processes are schematized in Fig 2a and 2b. As cohorts are identical, we are able to establish a causal relationship between changes in treatment strategy and increased survival time.

### Recapitulation of previous trial data

Using three identical cohorts, we evaluated patient outcomes when they received no treatment (Cohort 1), immunotherapy (Cohort 2), or oncolytic virus monotherapy (Cohort 3) to mimic the T-VEC OPTiM trial, where individuals were randomized to receive either intralesional T-VEC or subcutaneous GM-CSF [9].

In both the *in silico* immunotherapy and oncolytic virus monotherapy cases, the dosing schedules were identical to the ones used in OPTiM [9]: patients in the T-VEC arm received a priming dose of 10^6^ plaque forming units pfu/mL, followed by 10^8^ pfu/mL doses to a maximal total administration of 4 mL per treatment. T-VEC was administered every 14 days. Patients in the GM-CSF arm received 125 *μ*g/m^2^ of subcutaneous GM-CSF administered on 14 consecutive days followed by 14 days of no treatment. In both arms, treatment continued for up to 12 months but could be discontinued due to disease progression, intolerability, or the disappearance of injectable lesions. The median treatment length for the T-VEC and GM-CSF arms were 23 and 10 weeks respectively.

The amount of T-VEC administered in the OPTiM trial was patient and physician dependent [9]. In the simulated trial, we fixed an oncolytic virotherapy dose of 250 × 10^6^ virions, corresponding to roughly 1 × 10^6^ pfu [35]. Note that the units between the OPTiM trial [9] and our *in silico* trial differ owing only to the units of the mathematical model’s parameters and the conversion of pfu to infectious virions. Individuals receiving GM-CSF immunotherapy in the *in silico* trial were administered 125 *μ*g/m^2^ of GM-CSF daily for 14 days in 28 day cycles. For both arms, we simulated the model over a fixed treatment time of 6 months.

Late stage melanoma has a low survival rate [36]. Mortality as a function of tumour doublings has been estimated to occur between 40 and 45 tumour doublings [34, 37, 38]. Given that roughly 30 doublings occur before clinical presentation [38], we estimated that there are approximately 10 and 15 tumour doublings between diagnosis and death. *In silico* patients were therefore removed from the simulated trial after their predicted tumour size reached 2^λ^, where λ denotes the removal number of tumour doublings for each individual. For each individual, λ was obtained by sampling uniformly from the interval [10, 15], or the set of possible tumour doubling values between diagnosis and death. The incorporation of different disease stages within the OPTiM cohorts in our population approach is discussed in further detail in the Supplementary Information file S1 Text.

### Optimization routine for combined immuno- and oncolytic virotherapies

Adverse effects reported in the OPTiM trial including fatigue, chills and other flu like symptoms. Grade 3 adverse effects occurred in 36% and 21% of patients receiving T-VEC and GM-CSF, respectively. Our model does not specifically address adverse effects. To provide maximal therapeutic benefit with the lowest possible treatment burden, we defined individualized dosing regimens to be the schedule that minimizes the cumulative tumour burden (the area under the total tumour curve) over an individualization period of ten weeks and the cumulative dose (the total amount of therapy administered over the treatment time). Thus, we minimized the objective function

F(Dose)=CumulativeTumourBurden+αCumulativeDose,

where the positive scaling coefficient *α* weighted the importance of maximizing the therapeutic effect versus the need to minimize treatment burden. This weighting values takes the need for a treatment to be simultaneously effective and tolerable into account.

Tolerability of combined therapy was attained by bounding the permissible dose size to be four times the standard dose amount, consistent with the maximum dose for T-VEC in the OPTiM trial. In a clinical setting, it is preferable to administer discrete amounts of a drug, typically limited to be some multiple of the available vial size. We constrained the dose size to be 1–4-times the standard dose size for both immunotherapy and virotherapy. We allowed for daily immunotherapy dosing and restricted virotherapy administration to days 7, 14, 21, 28, 35, 42, 49, 56, 63, 70 so that virotherapy defined the beginning of a week-long treatment cycle. In total, 300 virtual patients underwent ten treatment cycles meaning there are 3000 total possible treatment cycles. Note that the schedule described above is potentially denser than what was administered by Andtbacka et al. [9]. We allowed for increased treatment frequency to measure its impact on improved clinical outcomes, under the constraint that the cumulative dose administered in the optimal treatment regimen must be less than the cumulative dose administered during the OPTiM trial [9].

To determine personalized dosing regimens, the optimal function *F*(Dose) was minimized over a ten-week treatment period using Matlab’s genetic algorithm function *ga* [39]. Genetic algorithms are heuristic global optimization routines inspired by natural selection [40–42] that are frequently employed to estimate parameters in computational biology models. They have also previously been applied to study optimal dosing routines in immunology [42].

We then generated personalized schedules for each of the 300 individuals in the optimal combination cohort. These schedules determined an empirical distribution of the probability of administering a dose of either immuno- or virotherapy on a given day of the treatment period. Sampling from this distribution, we next determined the probability that immunotherapy (*P*_*I*_(Day_*i*_)) or virotherapy (*P*_*V*_(Day_*i*_)) is administered on Day *i* of therapy to determine a probabilistic treatment schedule that replicated the results of the treatment optimization on the population-level (Fig 2b).

### Inference and validation of optimal treatment schedule

We first determined whether a dose of immunotherapy was to be administered on the *i*-th day of treatment by sampling from a Bernoulli distribution with probability given by *P*_*I*_(Day_*i*_) (see Table 1). If the sampling returns a success, a dose of immunotherapy was administered. To determine the size of the immunotherapy dose, we sampled from the empirical distribution inferred from the optimization step given in Table 1 (i.e. the probability of giving a dose of size *n* given that immunotherapy is administered on day *i*). If the *i*-th day is the beginning of a new treatment cycle, then virotherapy may be administered (Fig 2c). Whether a dose of oncolytic virus is administered and, if so, the dose size is determined in precisely the same way as for immunotherapy, using the empirical distribution for virotherapy given in Table 2.

**Table 1:** Inferred probability distribution for GM-CSF scheduling. The probability of administering immunotherapy (*P*_*I*_(Day_*i*_)) in each day of the treatment cycle and the conditional probability *P*_*I*_(*n*|Day_*i*_) of administering *n* doses of immunotherapy for *n* = 1, 2, 3, 4.

Day_*i*_	-3	-2	-1	Start of Cycle	1	2	3
*P*_*I*_(Day_*i*_)	0.2043	0.2020	0.2037	0.2027	0.2200	0.2047	0.2057
*P*_*I*_(1|Day_*i*_)	0.3719	0.3762	0.3519	0.3799	0.3803	0.3583	0.3387
*P*_*I*_(2|Day_*i*_)	0.1599	0.1419	0.1702	0.1694	0.1439	0.1482	0.1929
*P*_*I*_(3|Day_*i*_)	0.1550	0.1733	0.1637	0.1217	0.1348	0.1678	0.1378
*P*_*I*_(4|Day_*i*_)	0.3132	0.3086	0.3142	0.3289	0.3409	0.3257	0.3306

**Table 2:** Inferred probability distribution for T-VEC scheduling. The probability of administering virotherapy on each 7th day of the treatment cycle (*P*_*V*_(Day_7_)) and the conditional probability *P*_*V*_(*n*|Day_7_) of administering *n* doses of virotherapy for *n* = 1, 2, 3, 4.

**P_V_**(**Day_7_**)	**0.5487**
*P*_*V*_(1|Day_7_)	0.1592
*P*_*V*_(2|Day_7_)	0.1200
*P*_*V*_(3|Day_7_)	0.1597
*P*_*V*_(4|Day_7_)	0.5611

To test the effectiveness of the probabilistic dosing schedule, we created and cloned 200 new virtual patients, and separated them into three trial arms. The first cohort received the combined immuno- and virotherapy of 125 *μ*g/m^2^ of GM-CSF daily for 14 days in 28 day cycles and 1 dose of virotherapy every 14 days corresponding to a combination of the standard of care reported in the OPTiM trial [9]. A maintenance therapy schedule was derived from the results of the therapy optimization and was followed for the second cohort (see Results). Finally, the probabilistic dosing regimen determined from the population optimization was applied to the third arm. In all three arms, virtual patients received treatment for the median treatment duration of the OPTiM trial. Mortality and removal from the trial followed the same procedure described in the *Model Calibration* section above.

## Results

### Computational biology model successfully predicts existing therapy results

**Fig 3:**
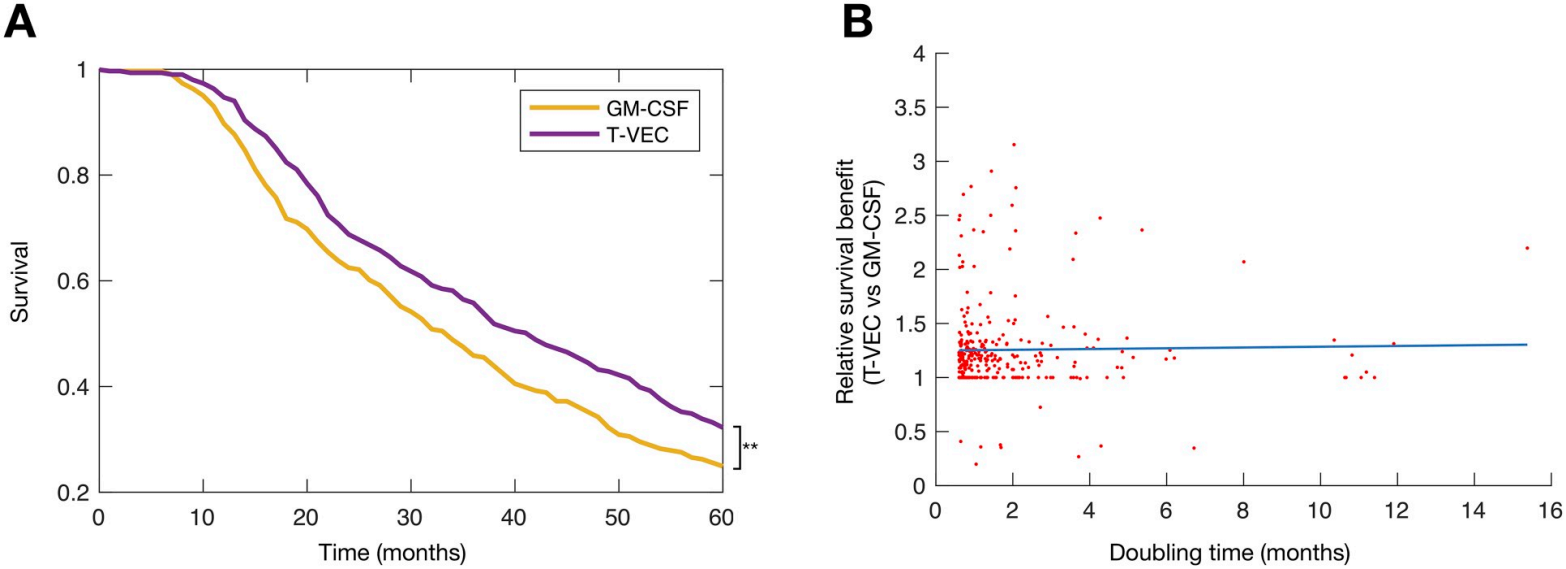
Treatment with oncolytic virus provides improved outcomes over immunotherapy in virtual clinical trial. **A)** Kaplan-Meier curves for patients in the immunotherapy and virotherapy arms of the virtual trial; **B)** The relative survival benefit for identical virtual patients. The ratio of survival time on T-VEC against survival time on GM-CSF for identical virtual patients (line of best fit, slope = 0.0035) establishes a causal relationship between treatment type and survival time, indicating that oncolytic virus therapy provided slightly larger survival gains in those with longer doubling times when compared to GM-CSF.

We first compared the model predictions to the OPTiM results [9] to evaluate the computational biology model’s ability to accurately represent the outcomes for patients receiving either GM-CSF or the oncolytic virus monotherapy T-VEC (Fig 3A) [9, 11]. No untreated virtual patient survived to the end of the trial (S2 Fig) and both of the treated cohorts display increased survival when compared to no treatment. Patients receiving virotherapy were the most likely to survive until the end of the *in silico* trial, with a median survival time of 39.0 months, as compared to the reported median overall survival time of 41.1 months for patients with stage IIIB, IIIC, or IVM1a melanoma in OPTiM. The median survival time for patients in the GM-CSF arm of the *in silico* clinical trial was 31.3 months, just outside of the 95% confidence interval of 17.4 to 29.6 months of the OPTiM trial. In both OPTiM and our *in silico* clinical trial, the null hypothesis that T-VEC and GM-CSF have the same efficacy was rejected with *p* < 0.001 using a log-rank test.

We considered the time from beginning of treatment until the tumour contains twice the initial number of tumour cells as the time from treatment initiation to failure. The median time to treatment failure was then predicted to be 2.9 (OPTiM trial: 2.9 with 95% confidence interval of 2.8-4.0) and 13.9 months (OPTiM trial: 8.2 with 95% confidence interval of 6.5-9.9) in the GM-CSF and T-VEC arms, respectively.

The relative treatment benefit of virotherapy vs. immunotherapy was established by ordering virtual patients according to their untreated tumour doubling time (Fig 3B), with longer doubling time indicating slower disease progression and less aggressive disease. A line of best fit with positive slope suggests that oncolytic virus therapy provided larger survival gains in those with longer doubling times when compared to GM-CSF, consistent with the increased survival fraction of patients with stage 3 melanoma in Figure 4(f) of Andtbacka et al. [9].

### “All or nothing” virotherapy dosing strategy

**Fig 4:**
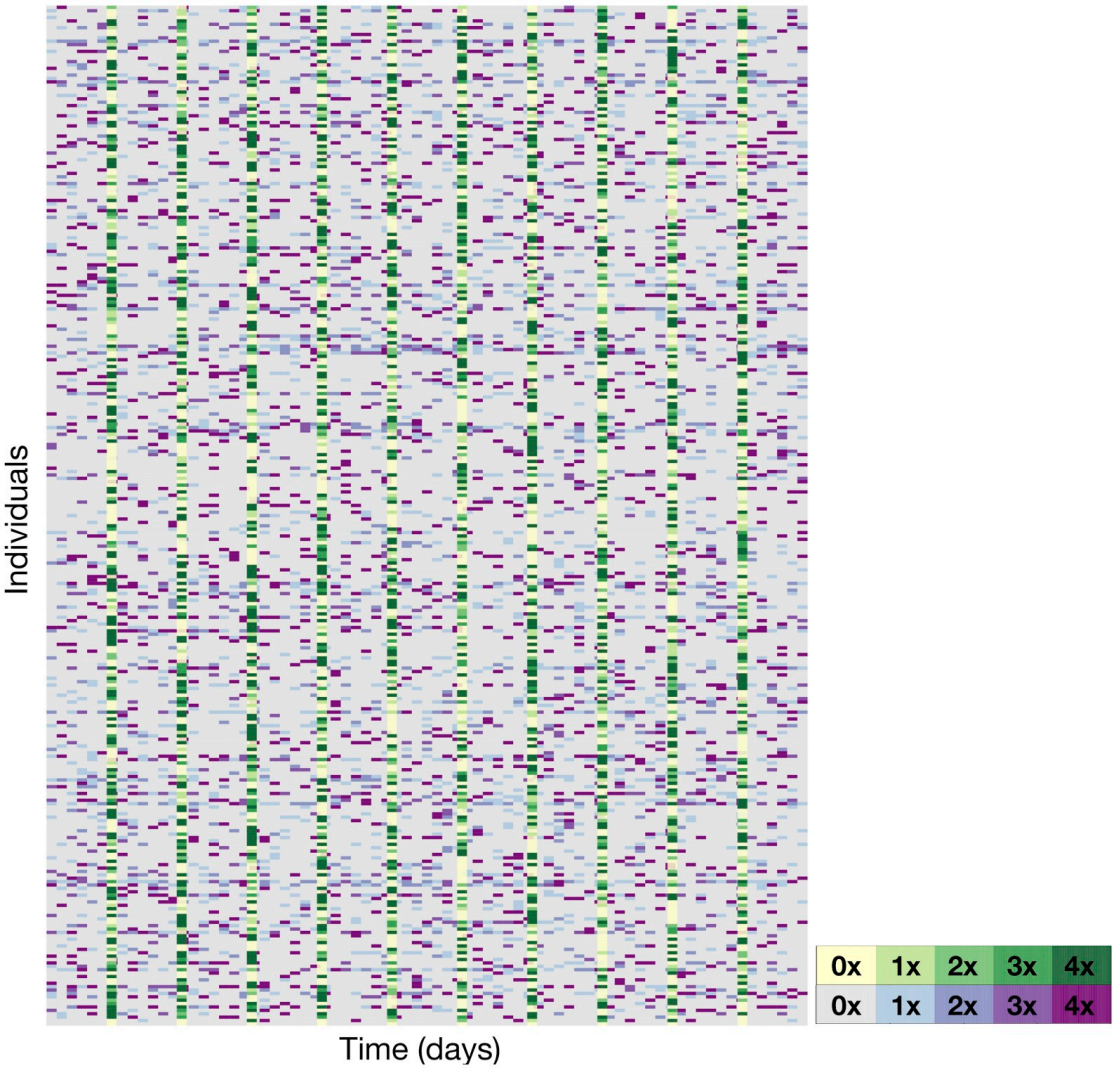
Optimal personalized dose scheduling for each of the 300 virtual patients. Dose size presented as a multiple of the standard dose with immunotherapy in shades of purple, and virotherapy in shades of green. The *n*th horizontal row corresponds to the *n*th virtual patient, while the *m*-th vertical column corresponds to the dose administered on day *m*.

We expected that treatment with GM-CSF would be used to either prime the immune system before virotherapy, or to support the immune response directly following administration of the oncolytic virus. However, as seen in Fig 4, no structure is easily discerned. To better understand the the structure of the underlying distribution of the individualized treatment schedules, we used the 3000 optimized treatment cycles (300 patients times 10 cycles) as a sample to define an empirical distribution of individualized treatment schedules. Then, from this empirical distribution we calculated the probability that any immunotherapy would be administered on each of the seven treatment cycle days of the optimized therapy regimen, as described in the Methods section (*Optimization Routine for Combined Immuno- and Oncolytic Virotherapy*). If a dose was given, we computed the conditional probability of administering a dose of one, two, three or four multiples of the standard dose (Table 1). We found that the probability of administering a dose of immunotherapy for a given treatment day is roughly constant at 20% throughout the treatment cycle. Interestingly, our results indicate that the immunotherapy dose given is expected to be either the smallest or the largest permitted, suggesting that immunostimulation is most useful as an additional instigator of immune recruitment when virotherapy does not elicit a sufficient immune response, or to otherwise maintain the immune response initiated by successful viral infection and lysis.

Contrary to the mono-immunotherapy dosing schedule, the conditional probabilities *P*_*V*_(Day_*i*_) for viral dose size are heavily skewed to the maximal tolerable dose (Table 2). Given that we assumed that oncolytic viruses induce anti-tumour activity through direct infection of tumour cells and secondary stimulation of an anti-tumour immune response, administering a large dose of oncolytic virus will improve outcomes in our model. Put differently, an “all or nothing” approach of dosing infrequently, but for maximal therapeutic benefit, is optimal, in contrast to the logic of the immunotherapy case.

These results suggest that administering immunotherapy between administrations of virotherapy serves mainly to maintain immune recruitment [43]. To test this hypothesis, we defined Maintenance Therapy to be the administration of virotherapy once every 14 days with immunotherapy administered evenly throughout on days 3, 6, 9, and 12 of each virotherapy treatment cycle. Dose size was calculated based on the cumulative expected weekly dose of immunotherapy (8 doses over 14 days) from the optimized regimen. Two doses of immunotherapy were therefore administered on days 3, 6, 9, and 12 to replicate the total expected immunotherapy dose. The same procedure was used to determine virotherapy doses.

### Maintenance and probabilistic combination therapies improve virtual patient survival

**Fig 5:**
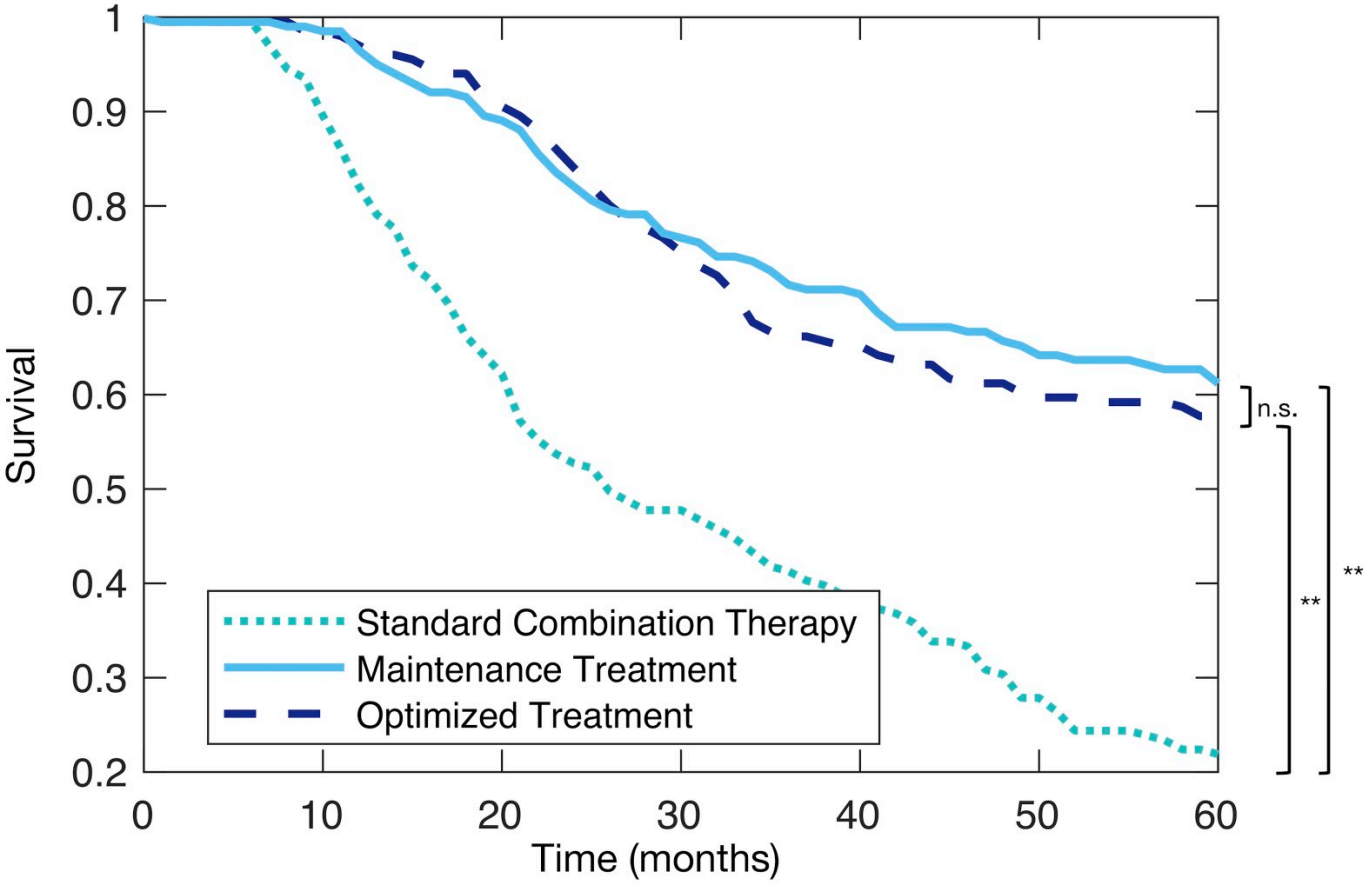
*In silico* clinical trial predicts improved outcomes for both probabilistic dosing strategies and maintenance therapy versus standard combination therapy. Kaplan-Meier curves for Arm 1: patients receiving Standard Combination Therapy (dotted turquoise line), Arm 2: Maintenance Treatment (solid light blue line), Arm 3: Probabilistic dosing regimen determined through the *in silico* clinical trial (dashed dark blue line).

Despite the shorter treatment period, both the maintenance and probabilistic combination immuno- and oncolytic virotherapies improved overall survival times as compared to the simulated OPTiM trial (Fig 5). Maintenance therapy similarly significantly increased mean survival time against mono-virotherapy (47.5 months vs. 35.36 months, two-sided t-test p-value of 1.02 × 10^−6^). The maintenance therapy and optimal dosing regimens also outperformed the standard combination therapy: on average, the mean survival time for patients receiving standard combination therapy was 26.1 months, while patients receiving the maintenance therapy or probabilistic dosing survived for 47.5 or 46.6 months respectively (two-sided t-test p-values of *p* < 0.001 in both cases). The hypothesis that the two treatments were equally efficacious was rejected with *p* < 0.001 using a log-rank test.

Minimizing the number of treatment days provides an additional measure of therapy tolerability. In the standard combination schedule, patients received 2 administrations of virotherapy and 14 doses of immunotherapy per 28 day cycle, requiring 15 total days of drug administration per 28 day cycle. The maintenance therapy schedule required a total of 11 treatment days per 28 day cycle (9 administrations of immunotherapy and 2 administrations of virotherapy), whereas patients given the optimized treatment schedule were administered an expected 5 immunotherapy doses and 2 virotherapy doses per 28 day cycle, for a total of 9 expected treatment days.

Crucially, the results of the individualized therapy can be translated into a clinically-actionable therapeutic strategy that significantly improves simulated clinical outcomes (maintenance schedule). Mean survival times between patients receiving the maintenance therapy and the probabilistic therapy were not significantly different (47.9 months vs 46.7 months, two-sided t-test p-value of 0.754). While for a given patient, improved outcomes from optimized and individualized regimens may be expected, leveraging the insights gained from the individualized cohort to produce population-wide improvements on a new cohort is a compelling achievement of our approach.

In summary, in terms of both end-points and dosing burden, immune maintenance therapy outperforms the standard-of-care combination therapy. Given the equivalent mean survival times between the maintenance and probabilistic schedules, our results also motivate rational therapy scheduling via *in silico* clinical trials to better establish the key mechanisms regulating treatment success prior to clinical trial enrolment.

## Discussion

Improving patient end-points and decreasing the drug burden during anti-cancer treatment are crucial components of cancer care. The introduction of new and advanced therapy modalities is critical to this goal. The approval of T-VEC, the first FDA approved, genetically modified oncolytic virus, was an important step forward for the treatment of late-stage melanoma that significantly improved patient survival over mono-immunotherapy GM-CSF administration. However, the question of whether combined immunotherapy and virotherapy will provide further benefits for patients and, if so, the optimal strategy for such combination therapy, remains. Running clinical trials is an expensive and onerous process. Trial failures are disappointing for patients, clinicians, and researchers, and contribute to overall attrition along the drug development pipeline. Here we have outlined a rational approach to therapy optimization that has significant consequences for how we effectively design and implement clinical trials to maximize their success, and how we treat melanoma with combined immuno- and virotherapy.

Leveraging our previous computational biology model, we developed an *in silico* clinical trial by creating virtual individuals based on a realistic distribution of model parameter values. Each generated individual was cloned and assigned to different trial cohorts. This innovative strategy enabled us to analyze the effects of distinct therapy procedures on the *same* person, something which is clearly infeasible in the real world. Personalization of treatment regimens was achieved by simultaneously minimizing cumulative tumour and drug burdens. A probabilistic dosing regimen was subsequently defined based on the resulting personalized treatment schedules. Incorporating clinical realities, we determined that standard combination therapy was improved upon by both a maintenance strategy (where immunotherapy is administered evenly throughout each virotherapy cycle) and this probabilistic dosing strategy. It is worth noting that the maintenance type therapy performed equivalently in terms of endpoints than the optimized scheduling, illustrating the utility of model-based optimization techniques in identifying and developing improved, clinically-actionable therapeutic strategies.

There are differences between OPTiM and our *in silico* trial. First, while we can broadly recreate the number of individuals in each stage of disease, we cannot identically reproduce the underlying distribution of patients. Accordingly, our results are highly dependent on the virtual patients selected for participation based on their tumour doubling time, and would be improved through the incorporation of detailed staging and patient distribution data. Second, the administration of an oncolytic virus can lead to an anti-viral adaptive immune response and a decrease in treatment efficacy that is currently not accounted for in the model. Our approach does not currently address therapy side effects, however addressing the development of immune tolerance to OVs is an area of future investigation. Last, our computational model simplifies tumour-immune interactions by consolidating all immune cells into a single phagocyte population. We also considered a single cytokine as a cipher for all pro-inflammatory responses induced by tumour-immune communication. We believe that these considerations do not significantly impact on our general results, but they should be addressed in future work to increase the precision of the predicted personalized regimens. Ideally, empirically determined distributions for the model’s parameter values would be available to strengthen the model’s predictions. Fortunately, notwithstanding the general unavailability of such data, our parameterization successfully recapitulated the OPTiM trial results.

Despite these limitations, our results underline the contribution of computational biology to understanding the determinants of improved clinical care and support continued efforts towards rational therapy design. Significantly, this computational biology study suggests promising avenues of investigation towards tailored combination immunotherapy/oncolytic virotherapy for patients with late-stage melanoma.

## Supporting information

S1 Text Supplementary information file. (PDF)

S1 Fig Parameter fitting results. A and B) Data (red circles) from Dingli et al. [31] for tumour growth in immunocompromised mice compared to model predictions (solid black lines). C) Comparisons of model predictions (solid black lines) and the Toda et al. data [32] (red circles) for the number of viable cells following the administration of T-VEC.
(TIFF)

S2 Fig Survival outcomes of untreated individuals. No untreated individual survives to end of 60 month trial.
(TIFF)

S3 Fig Local parameter sensitivity analysis. Left: dependence of tumour burden on the parameters shown on the y-axis. Right: dependence of tumour doubling time on the parameters shown on the y-axis. In both cases, parameters were varied by ±10%. Tumour doubling times of Inf indicate that the tumour did not reach twice the initial size.
(TIFF)

S4 Fig Individualized parameter distributions are normal. The distributions of the computational biology’s parameters for the 300 *in silico* individuals in the optimization trial were confirmed to be normal by the Shapiro-Wilk test. Dark blue: determined to be normal at *α* = 0.05 significance level; grey: weakly normal at *α* = 0.05 significance level. p-values (indicated on each graph) greater than 0.05 imply no statistically significant difference between parameter distribution and the normal distribution.
(TIFF)

S1 Table Mean parameter estimates. The vector **p** (see main section *Generation of in-silico individuals and patient cohorts*) with biological interpretations. See Cassidy and Humphries [28] for detailed descriptions of each parameter.
(PDF)
