# Supplementary material for: Determinants of combination GM-CSF immunotherapy and oncolytic virotherapy success identified through in silico treatment personalization
Source: PLoS Comput Biol. 2019 Nov 27;15(11):e1007495. doi: 10.1371/journal.pcbi.1007495 (PMC6880985; doi:10.1371/journal.pcbi.1007495)
Supplement: S1 Text — (PDF) [file pcbi.1007495.s001.pdf]

Determinants of combination GM-CSF immunotherapy and oncolytic virotherapy success identified through *in silico* treatment personalization

Tyler Cassidy, Morgan Craig

The computational biology model (described textually in Eq. (S1)) was based on the model of Cassidy and Humphries [1] and explicitly included heterogeneity in tumour cell reproduction velocity and tumour-immune interactions via a distributed delay differential equation. The model describes both quiescent and G_1 phase tumour cell populations while modelling the remainder of mitosis as a delayed process, and incorporates a phagocyte population and a proinflammatory cytokine that drives the tumour-immune interaction through increased phagocyte recruitment.

Let $Q(t)$ and $G_1(t)$ denote the quiescent and proliferative phase susceptible tumour cells, respectively, $C(t)$ the concentration of GM-CSF, $P(t)$ the phagocyte concentration in the tumour microenvironment, $V(t)$ the concentration of oncolytic virions, and $I(t)$ the number of infected tumour cells. Infection of susceptible tumour cells occurred at rate η, while tumour-immune interactions took place with rate $\psi_{Q,G}$.

To account for immune selection, we included a resistant strain of tumour cells undetectable to the immune system (represented by $Q_R(t)$, $G_{1,R}(t)$, analogous to the susceptible population). We assumed that tumour cells successfully completing mitosis could randomly mutate into the immune resistant strain with probability $\mu = 1 \times 10^{-10}$, and we assumed that the mutated strain of cancer cells reproduced identically to the non-mutated strain. For convenience, we denote the state vector at time t by
$U(t) = [Q(t), G_1(t), I(t), V(t), P(t), C(t), Q_R(t), G_{1,R}(t)]$.

$$\left.\begin{aligned}
\frac{\mathrm{d}}{\mathrm{dt}}Q(t) &= \text{Mitotic Output} - \text{Transit to active phase} \\
&\quad -\text{Apoptosis} - \text{Immune Death} \\
\frac{\mathrm{d}}{\mathrm{dt}}G_1(t) &= \text{Transit from quiescence} - \text{Transit to Mitosis} \\
&\quad -\text{Apoptosis} - \text{Viral Infection} - \text{Immune Death} \\
\frac{\mathrm{d}}{\mathrm{dt}}I(t) &= \text{Viral infection} - \text{Death of infected cells} \\
\frac{\mathrm{d}}{\mathrm{dt}}V(t) &= \text{Dose}_V(t) + \text{Lysis} - \text{Viral infection} - \text{Virion death} \\
\frac{\mathrm{d}}{\mathrm{dt}}P(t) &= \text{Immune recruitment} - \text{Phagocyte death} \\
\frac{\mathrm{d}}{\mathrm{dt}}C(t) &= \text{Dose}_C(t) + \text{Cytokine Production} - \text{Renal clearance} \\
\frac{\mathrm{d}}{\mathrm{dt}}Q_R(t) &= \text{Development of Resistance} + \text{Mitotic Output} \\
&\quad -\text{Transit to active phase} - \text{Apoptosis} \\
\frac{\mathrm{d}}{\mathrm{dt}}G_{1,R}(t) &= \text{Transit from quiescence} - \text{Transit to Mitosis} \\
&\quad -\text{Apoptosis} - \text{Viral Infection.}
\end{aligned}\right\} \tag{S1}$$

The differential equations describing the progression of disease are

$$\left.\begin{aligned}
\tfrac{\mathrm{d}}{\mathrm{dt}}Q(t) &= 2(1-\mu)\int_{-\infty}^{t}\exp\left[-\int_{\sigma}^{t}\hat{d}_K+\eta(U(x))+\psi_G(U(x))\mathrm{d}x\right]a_2G_1(\sigma)K(t-\sigma)\mathrm{d}\sigma \\
&\quad -\left[a_1+d_1+\psi_Q(U(t))\right]Q(t) \\
\tfrac{\mathrm{d}}{\mathrm{dt}}G_1(t) &= a_1Q(t)-\left[a_2+d_2+\eta(V(t))+\psi_G(U(t))\right]G_1(t) \\
\tfrac{\mathrm{d}}{\mathrm{dt}}Q_R(t) &= 2\mu\int_{-\infty}^{t}\exp\left[-\int_{\sigma}^{t}\hat{d}_K+\eta(U(x))+\psi_G(U(x))\mathrm{d}x\right]a_2G_1(\sigma)K(t-\sigma)\mathrm{d}\sigma \\
&\quad -\left[a_1+d_1\right]Q_R(t) \\
&\quad +2\int_{-\infty}^{t}\exp\left[-\int_{\sigma}^{t}\hat{d}_K+\eta(U(x))\mathrm{d}x\right]a_2G_{1,R}(\sigma)K(t-\sigma)\mathrm{d}\sigma \\
\tfrac{\mathrm{d}}{\mathrm{dt}}G_{1,R}(t) &= a_1Q_R(t)-\left[a_2+d_2+\eta(V(t))\right]G_{1,R}(t) \\
\tfrac{\mathrm{d}}{\mathrm{dt}}I(t) &= \eta(V(t))\left[G_1(t)+G_{1,R}(t)+N(t)\right]-\delta I(t) \\
\tfrac{\mathrm{d}}{\mathrm{dt}}V(t) &= \mathrm{Dose}_V(t)-\eta(V(t))\left[G_1(t)+G_{1,R}(t)+N(t)\right]+\alpha[\delta I(t)]-\omega V(t) \\
\tfrac{\mathrm{d}}{\mathrm{dt}}C(t) &= \mathrm{Dose}_C(t)+C_{prod}(U(t))-k_{elim}C(t) \\
\tfrac{\mathrm{d}}{\mathrm{dt}}P(t) &= \varphi(C(t))-\gamma_pP(t).
\end{aligned}\right\} \tag{S2}$$

Phagocytosis of quiescent and mitotic tumour cells occurs with respective rate

$$\psi_Q(U(t))Q(t)=\frac{k_pP(t)}{1+k_qQ(t)}Q(t),\quad \text{and}\quad \psi_G(U(t))G_1(t)=\frac{k_pP(t)}{1+k_sG_1(t)}G_1(t).$$

As in Cassidy and Humphries, we enforced $k_q=k_s$. The infection of susceptible cells by the oncolytic virus was modelled similarly by

$$\eta(V(t))=\kappa\frac{V(t)}{\eta_{1/2}+V(t)},$$

where $\eta_{1/2}$ represents the half effect concentration of infectious virions and κ is the maximum infection rate. We assumed that phagocytes are recruited to the tumour microenvironment by GM-CSF at rate

$$\varphi(C(t))=\frac{k_{cp}C(t)}{C_{1/2}+C(t)}.$$

Finally, GM-CSF was produced at the basal rate of C^*_{prod}. Infection and phagocytosis of tumour cells drives increased GM-CSF production according to

$$(C^{max}_{prod}-C^*_{prod})\frac{[\delta I(t)+\Psi(U(t))]}{\Psi_{1/2}+[\delta I(t)+\Psi(U(t))]}$$

where C^{max}_{prod} is the maximal production rate of GM-CSF. The full equation of GM-CSF production is then modelled by

$$C_{prod}(U(t))=C^*_{prod}+(C^{max}_{prod}-C^*_{prod})\frac{[\delta I(t)+\Psi(U(t))]}{\Psi_{1/2}+[\delta I(t)+\Psi(U(t))]}.$$

The total number of cells in the cell cycle is given by

$$N(t) = \int_0^\infty a_2 \exp\left[-\int_{t-\xi}^t \hat{d}_K + \eta(U(x))\mathrm{d}x\right]\left(1 - \int_0^\xi K(\sigma)\mathrm{d}\sigma\right) \times \left(G_1(t-\xi)\exp\left[-\int_{t-\xi}^t \psi_G(U(x))\mathrm{d}x\right] + G_{1,R}(t-\xi)\right)\mathrm{d}\xi.$$

We modelled the subcutaneous administration of N doses of GM-CSF similar to Craig et al.[2] by

$$\mathrm{Dose}_C(t) = \sum_{i=1}^N \frac{k_a^c F_c \mathrm{Admin}_i^c(t)}{Vol}\exp\left[-k_a^c(t-t_i)\right], \tag{S3}$$

where the amount of GM-CSF administered at time t_i is Dose_i^c and

$$\mathrm{Admin}_i^c(t) = \begin{cases} 0 & \text{if} \quad t < t_i \\ \mathrm{Dose}_i^c & \text{if} \quad t \geqslant t_i. \end{cases}$$

The parameter k_a^c denotes the absorption rate of GM-CSF, and F the bioavailable fraction of GM-CSF. Similarly, the intralesion administration of oncolytic viruses [3, 4, 5] was modelled as

$$\mathrm{Dose}_V(t) = \sum_{j=1}^N \frac{k_a^v F_v \mathrm{Admin}_i^v(t)}{Vol}\exp\left[-k_a^v(t-t_j)\right] \tag{S4}$$

where Dose_j^v is the amount of virus administered at time $t = t_j$ and

$$\mathrm{Admin}_j^v(t) = \begin{cases} 0 & \text{if} \quad t < t_j \\ \mathrm{Dose}_j^v & \text{if} \quad t \geqslant t_j. \end{cases}$$

As we considered intralesional administration, $F_V = 1$. Further, absorption into the tumour was assumed to be much faster than cytokine absorption, so that $k_a^v >> k_a^c$.

Equation (S2) is a distributed delay differential equation with delay kernel $K(t)$. This delay represents the duration of the mitotic portion of the cell cycle. Here, we assumed that the cell cycle duration is gamma distributed (other distributions are also possible [1, 6]). Model predictions were obtained using the stiff ODE solver ode15s in Matlab after applying the technique developed by Cassidy and Humphries [1] to reduce (S2) to the equivalent finite dimensional system of ODEs given by (S5). The linear chain technique consists of replacing the distributed delay term in (S2) by the solution of a system of linear ordinary differential equations (ODEs). This system of ODEs introduces two new parameters, k_{tr} and j, that are

determined by the parametrization of the gamma distribution $K(t)$ in (S2).

$$\left.\begin{array}{rcl}
\frac{\mathrm{d}}{\mathrm{dt}}Q(t) & = & 2(1-\mu)k_{tr}A_j(t) - a_1Q(t) - d_1Q - \psi_Q(U(t))Q(t) \\
\frac{\mathrm{d}}{\mathrm{dt}}G_1(t) & = & a_1Q(t) - a_2G_1(t) - d_2G_1(t) - \psi_G(U(t))G_1(t) - \eta(V(t))G_1(t) \\
\frac{\mathrm{d}}{\mathrm{dt}}A_1(t) & = & a_2G_1(t) - k_{tr}A_1(t)) - [\hat{d}_g + \eta(V(t)) + \psi_G(U(t))]A_1(t) \\
\frac{\mathrm{d}}{\mathrm{dt}}A_i(t) & = & k_{tr}(A_{i-1}(t) - A_i(t)) - [\hat{d}_g + \eta(V(t)) + \psi_G(U(t))]A_i(t) \quad \text{for} \quad i = 2, 3..., j \\
\frac{\mathrm{d}}{\mathrm{dt}}Q_R(t) & = & 2\mu k_{tr}A_j(t) + 2k_{tr}A_{j,R}(t) - a_1Q_R(t) - d_1Q_R(t) \\
\frac{\mathrm{d}}{\mathrm{dt}}G_{1,R}(t) & = & a_1Q_R(t) - a_2G_{1,R}(t) - d_2G_{1,R}(t) - \eta(V(t))G_{1,R}(t) \\
\frac{\mathrm{d}}{\mathrm{dt}}A_{1,R}(t) & = & a_2G_{1,R}(t) - k_{tr}A_{1,R}(t)) - [\hat{d}_g + \eta(V(t)) + \psi_G(U(t))]A_{1,R}(t) \\
\frac{\mathrm{d}}{\mathrm{dt}}A_{i,R}(t) & = & k_{tr}(A_{i-1,R}(t) - A_{i,R}(t)) - [\hat{d}_g + \eta(V(t)) + \psi_G(U(t))]A_{i,R}(t) \quad \text{for} \quad i = 2, 3..., j \\
\frac{\mathrm{d}}{\mathrm{dt}}I(t) & = & -\delta I(t) + \eta(V(t))\,[G_1(t) + N(t)] \\
\frac{\mathrm{d}}{\mathrm{dt}}V(t) & = & \alpha\delta I(t) - \omega V(t) - \eta(V(t))\,[G_1(t) + N(t)] \\
\frac{\mathrm{d}}{\mathrm{dt}}C(t) & = & C_{prod}(U(t)) - k_{elim}C(t) \\
\frac{\mathrm{d}}{\mathrm{dt}}P(t) & = & \varphi(C(t)) - \gamma_p P(t)
\end{array}\right\} \tag{S5}$$

Initial conditions were carefully chosen to ensure that the solution of the finite dimensional (S5) defined a solution of (S2) [1, 6].

Model parametrization

To begin, we converted tumour volumes to cell numbers by assuming that $1\,\mathrm{mm}^3 = 1 \times 10^6$ cells [7]. To accurately account for the heterogeneity in cell cycle time, we integrated the intermitotic time of cervical cancer cells measured by Sato et al. [8]. There, the intermitotic time was determined by calculating the division time of a HeLa parent cell and then tracking the two daughter cells until their division. For each daughter cell, the intermitotic time was given by the time difference between birth and division, with a mean duration of 1.40 (standard deviation of $s = 0.28$ days). This measurement also includes the G_1 phase of the cell cycle. In (S2), cells that divide spend, on average, $1/a_2$ days in G_1. Thus, the mean of the delay kernel in (S2), τ, satisfied

$$\tau = 1.40 - 1/a_2, \tag{S6}$$

which provided a lower bound on the value of a_2. The gamma distribution has a density given by

$$g^j_{k_{tr}}(t) = \frac{k^j_{tr}}{\Gamma(j)} t^{j-1} \exp\left[-k_{tr}t\right]$$

with mean $\tilde{t} = j/k_{tr} = \tau$ and variance $s^2 = \tau^2/j$. Starting from $s^2 = j/k^2_{tr}$, we multiplied by unity to obtain $s^2 = (j^2/k^2_{tr})/j$. Using the definition $\tau = j/k_{tr}$, we found $Var[t] = (\tau)^2/j$. This yielded $j = \tau^2/s^2$. Once the parameter j was determined, $\tau = j/k_{tr}$ yielded the

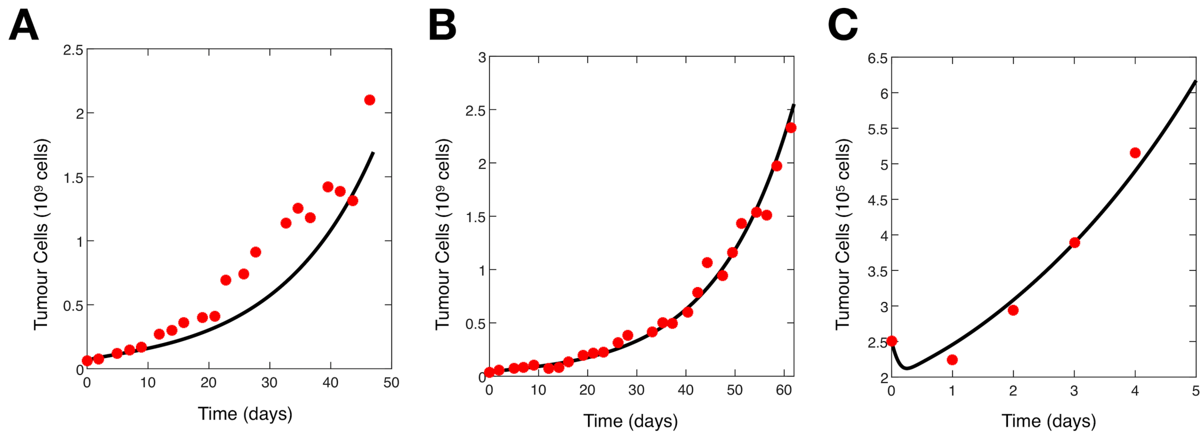

Figure S1: **Parameter fitting results.** A and B) Data (red circles) from Dingli et al. [7] for tumour growth in two different immunocompromised mice compared to model predictions (solid black lines). C) Comparisons of model predictions (solid black lines) and the Toda et al. data[9] (red circles) for the number of viable cells following the administration of T-VEC.

parameter k_{tr}, thus completing the parametrization of the delay kernel in (S2). To use the linear chain technique[1], we enforced that $j \in \mathbb{N}$.

Dingli et al.[7] administered myeloma cells to 6 week old irradiated and severely immunocompromised mice and measured the increase in tumour volume over 40+ days. We digitized the data from their Figure 1 representing tumour growth in two different mice, and used them to fit the tumour growth parameters a_1, a_2 and d_2 in the absence of immune pressure. To reduce the number of parameters to be estimated, we fixed the apoptosis rate of quiescent cells to be $d_1 = 0$. Since the mice in the Dingli et al.[7] study were immunocompromised and no viral therapy was administered, we only considered the reduced system of $Q(t), S(t)$ and $N(t)$ of (S2), and then calculated the distribution parameters using (S6) and $b = j/\tau$. As only the initial number of tumour cells (and not their distribution across the cell cycle) is known for each mouse, we distributed the initial inoculum of tumour cells across quiescent and the active portions of the cell cycle according to the proportion of time spent in each phase. Then, for each parameter set, we simulated the reduced mathematical model and minimized the least-squares error

$$err = \sum_{i=1}^{N} (y_i - \hat{y}_i)^2$$

between model predictions (y_i) and the data ($\hat{y}_i$) from Figures 1 (a) and (c) in Dingli et al. using the *fmincon* function in Matlab (Fig. S1 A and B).

Viral and infected cell kinetic parameters were determined using *in vitro* and *in vivo* studies of T-VEC kinetics and data from Toda et al. [9]. In their study, Toda et al. injected murine melanoma cells into mice and waited for the tumours reached 5 mm in size. At that point, defective HSV virus encoding for GM-CSF was injected into the tumour. The increase in tumour volume as compared with untreated control mice was measured over 15 days. To fit the Toda et al. data, we used the tumour growth parameters a_1, a_2, and d_2 from the Dingli et al. estimates while setting $d_1 = 0$, and simulated tumour growth during 15

days as a control simulation. Then, for fixed parameter set $[\kappa, \eta_{1/2}, \omega, \delta, \alpha]$, we simulated the administration of one dose of oncolytic virus (supplied in 1 mL vials with a concentration of 1×10^6 pfu/mL or 1×10^8 pfu/mL). We then normalized the treated simulation against the control simulation and fit to the Toda et al. data.

To reduce identifiability issues, we also integrated data from Randazzo et al. [10]. There, an oncolytic HSV virus was mixed with tumour cells *in vitro* and the proportion of viable cells was studied for different multiplicities of infection. As before, we fixed the tumour growth parameters and, for fixed parameter set $[\kappa, \eta_{1/2}, \omega, \delta, \alpha]$, simulated infection and calculated the percentage of viable (non-infected) cells. For each of the Toda et al. and Randazzo et al. datasets, we calculated the least-squares error between simulations and data and minimized the sums of their errors (Fig. S1 C).

Due to the lack of PopPK/semi-mechanistic models of GM-CSF, we adapted a model [2] for granulocyte colony stimulating factor (G-CSF), a similar protein, that includes both neutrophil mediated and renal clearance of circulating G-CSF. Since we were primarily interested in immune cells that do not have a G-CSF receptor (macrophages and T-cells), we did not consider neutrophil mediated clearance and fixed $k_{elim} = 0.16139$ 1/day. The basal concentration of GM-CSF in healthy patients is 2.43 pg/mL [11], given by

$$C^* = \frac{C^*_{prod}}{k_{elim}},$$

at homeostasis. Thus,

$$C^*_{prod} = k_{elim} C^* = 0.0003921777 \text{ng/mL/day}.$$

To calculate the maximal cytokine production, C^{max}_{prod}, we considered experimental data from Liu et al. [12] from the injection of carcinoma cells into mice. Therein, different MOIs of oncolytic HSV virus expressing GM-CSF was administered after a period of initial tumour growth. Throughout, circulating GM-CSF was measured through ELISA. To replicate the experimental set up, we simulated the administration of oncolytic virus in and minimized the least-squares error between the simulation and the experimental data for fixed parameter values $[C^{max}_{prod}, C_{1/2}]$, while holding the tumour growth and viral dynamics at their previously estimated values.

The parametrization of the immune compartment was based on Barish et al. [13], where a mathematical model of murine tumour growth in the presence of a competent immune system and dendritic cell vaccine was developed. The structure of the Barish et al. model of immune dynamics is quite similar to our differential equation for $P(t)$. Therefore, we used their clearance rate of T-cells as the clearance rate of phagocytes so $\gamma_p = 0.35$. Barish et al. also modelled the recruitment of phagocytes by dendritic cell activation. As activated dendritic cells produce pro-inflammatory cytokine, we adopted their phagocyte recruitment rate and set $k_{cp} = 4.6754$ [13]. The remaining immune involvement parameters, k_p and $k_q = k_s$, were set to ensure that the average patient had a tumour doubling time that fell near the center of the range found in patients with late stage melanoma [14]. Parameters values for an average patient (see main section *Generation of In Silico Individuals and Patient Cohorts*) are provided in Table S1.

Parameter	Value	Biological Interpretation (Unit)
a_1	1.183	Quiescent to interphase rate (1/day)
d_1	0	Quiescent death rate (1/day)
a_2	1.758	Interphase to active phase rate (1/day)
d_2	0.539	Interphase death rate (1/day)
$\hat{d}_g$	0.167	Active phase death rate (1/day)
κ	3.53	Virion contact rate (1/day)
$\eta_{1/2}$	0.51	Virion half effect concentration (virions)
δ	4.96	Lysis rate (1/day)
α	0.00829	Lytic virion release rate (virions/cell)
ω	9.686	Virion death rate (1/day)
k_{cp}	4.675	Maximal phagocyte production rate (10^{10} cells/day)
$C_{1/2}$	0.739	Phagocyte production half effect (ng/mL/day)
$\Psi_{1/2}$	5	Cytokine production half effect (10^{10} cells/day)
γ_p	0.35	Phagocyte death rate (1/day)
C^*_{prod}	3.92×10^{-4}	Homeostatic cytokine production rate (ng/mL/day)
C^{max}_{prod}	1.429	Maximal cytokine production rate (ng/mL/day)
k_{elim}	0.16139	Cytokine elimination rate (1/day)
k_p	0.05	Phagocyte-tumour cell contact rate (1/day)
$k_{q,s}$	10	Phagocyte cell digestion constant
τ	0.8354	Expected cell cycle duration (day)
j	9	Number of transit compartments in (S5)
k_{tr}	10.77	Transit rate in (S5)

Table S1: **Mean parameter estimates.** The parameters at left are all elements of the vector **p** representing an "average" virtual patient in the simulation of (S5) (see main section *Generation of in-silico individuals and patient cohorts*) with biological interpretations. See Cassidy and Humphries [1] for detailed descriptions of each parameter.

Virtual population patient distribution and history function derivation

The distribution of disease stage in the OPTiM trial was a crucial component for considering the overall survival statistics given the decreased 5 year survival rate for patients with more advanced disease. The original trial registered patients with not surgically resectable stage IIIB to IV melanoma. In the T-VEC cohort, 30% of patients had stage 3 melanoma. The median progression time from first treatment to lymph metastases was 18 months, while the median time to distant metastases was 24 months [15]. To reproduce this distribution of disease stage in our virtual population, for each of the 30% of virtual patients with stage 3 melanoma, we assumed that initial treatment was discontinued sometime s_0 in the past, where s_0 was sampled uniformly from the interval $[16, 20]$. Similarly, for the 70% of virtual patients with stage 4 melanoma, we sampled s_0 uniformly from $[22, 26]$.

To set the history function φ_G for (S2), we assumed that there was a tumour of size $T_0 > 0$ at cessation of the initial therapy. We began by modelling untreated tumour growth

by

$$\frac{\mathrm{d}}{\mathrm{dt}}\left[Q(s)+G_1(s)\right] \approx \frac{\mathrm{d}}{\mathrm{dt}}T(s) = \left[2a_2\exp[-d_3\tau] - a_2 - d_2\right]T(s), \quad s \in [s_0, 0].$$

Thus, at the beginning of treatment, the total number of tumour cells for each patient was

$$T_0 \exp\left[\left(2a_2\exp[-d_3\tau] - a_2 - d_2\right)s_0\right].$$

We distributed these tumour cells across the quiescent, G_1, and mitotic populations according to the expected fraction of time spent in each population.

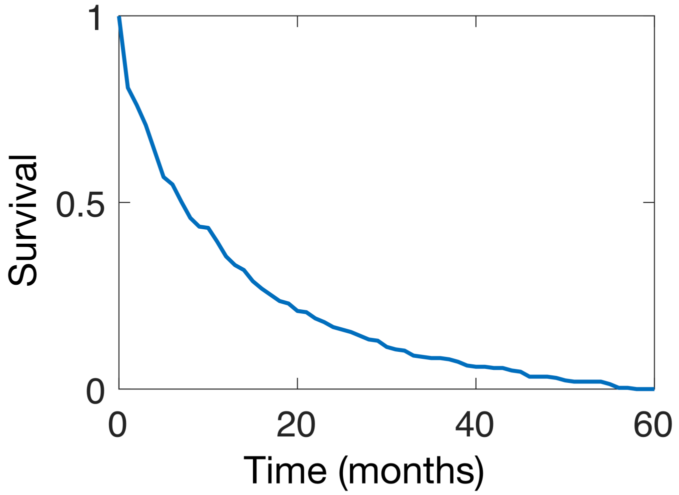

Figure S2: **Survival outcomes of untreated individuals.** No untreated individual survives to end of 60 month trial.

Local sensitivity analysis

Particularly important physiological processes in disease dynamics were identified by performing a sensitivity analysis on the mathematical model's parameters. Each parameter was varied one-by-one by 10%. The influence of each of these variations was measured by comparing the predicted tumour doubling time and the tumour burden after 15 months to the model's predictions without any parameter changes according to

$$\%\text{ change in tumour burden} = 100\% \times \frac{\text{Tumour burden with parameter change}}{\text{Tumour burden without parameter change}}.$$

The parameters controlling the dynamics of mitotic cells were the most sensitive, with the 10% change accounting for drastic changes in disease burden. For example, increasing the rate at which G_1 cells enter into mitosis results in a 60-fold increase in tumour burden, while decreasing this rate decreased the tumour burden by a factor of 33 (Figure S3), indicating that interventions that inhibit the specific transition from G_1 into mitosis may offer consequential therapeutic benefits.

	Tumour burden (% of normal)		Tumour doubling time (% of normal)	
	-10%	+10%	-10%	+10%
**Entry to G1**	91.99	107.9	91.36	106.4
**Entry to active phase**	3.454	6072	Inf	50.54
**Apoptosis: G1**	8120	4.159	46.8	Inf
**Cell cycle length**	110.7	90.21	120.7	78.72
**Maximal tumour/immune interaction rate**	100.9	99.09	99.69	100.3
**50% response: phagocytes**	100.9	99.09	99.69	100.3
**50% response: tumour cells**	98.99	100.8	100.3	99.72
**Maximal phagocyte production rate**	100.2	99.83	99.98	100

Colour scale: 50, 60, 70, 80, 90, 100, 110, 120

Figure S3: **Local parameter sensitivity analysis.** Left: dependence of tumour burden on the parameters shown on the y-axis. Right: dependence of tumour doubling time on the parameters shown on the y-axis. In both cases, parameters were varied by ±10%. Tumour doubling times of Inf indicate that the tumour did not reach twice the initial size.

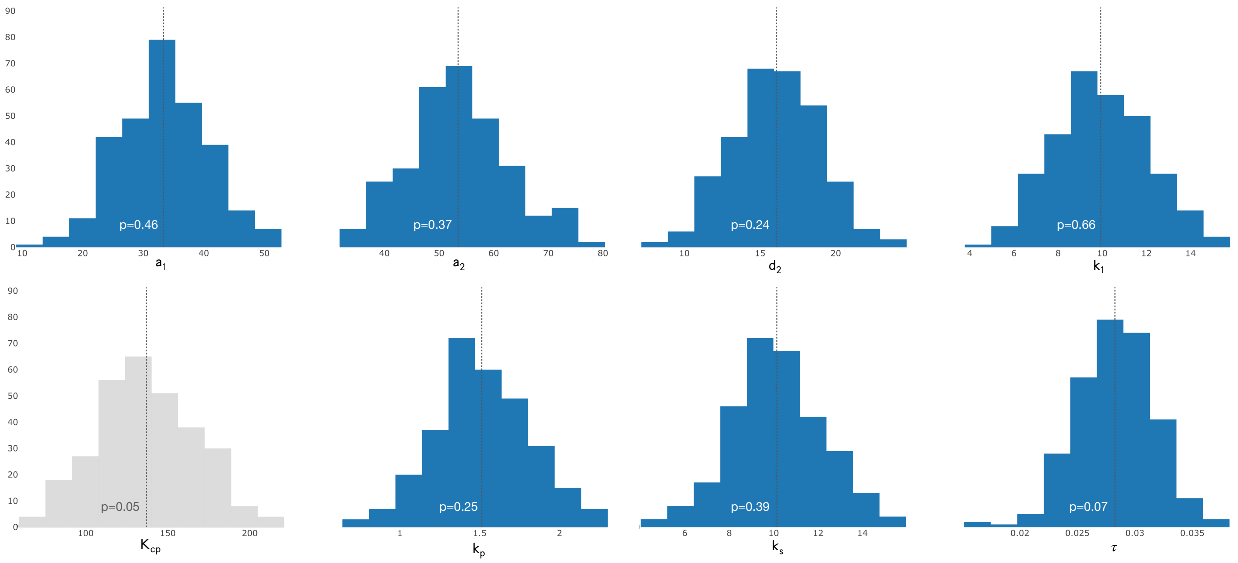

Figure S4: **Individualized parameter distributions are normal.** The distributions of the computational biology's parameters for the 300 *in silico* individuals in the optimization trial were confirmed to be normal by the Shapiro-Wilk test. Dark blue: determined to be normal at α=0.05 significance level; grey: weakly normal at α=0.05 significance level. p-values (indicated on each graph) greater than 0.05 imply no statistically significant difference between parameter distribution and the normal distribution.

References

[1] Cassidy T, Humphries AR. A mathematical model of viral oncology as an immuno-oncology instigator. Math Med Biol A J IMA. 2019;To appear. doi:10.1093/imammb/dqz008.

[2] Craig M, Humphries AR, Mackey MC. A mathematical model of granulopoiesis incorporating the negative feedback dynamics and kinetics of G-CSF/neutrophil binding and internalization. Bull Math Biol. 2016;78(12):2304–2357. doi:10.1007/s11538-016-0179-8.

[3] Andtbacka RHI, Kaufman HL, Collichio F, Amatruda T, Senzer N, Chesney J, et al. Talimogene laherparepvec improves durable response rate in patients with advanced melanoma. J Clin Oncol. 2015;33(25):2780–2788. doi:10.1200/JCO.2014.58.3377.

[4] Bommareddy PK, Patel A, Hossain S, Kaufman HL. Talimogene Laherparepvec (T-VEC) and Other Oncolytic Viruses for the Treatment of Melanoma. Am J Clin Dermatol. 2017;18(1):1–15. doi:10.1007/s40257-016-0238-9.

[5] Johnson DB, Puzanov I, Kelley MC. Talimogene laherparepvec (T-VEC) for the treatment of advanced melanoma. Immunotherapy. 2015;7(6):611–619. doi:10.2217/imt.15.35.

[6] Cassidy T, Craig M, Humphries AR. Equivalences between age structured models and state dependent distributed delay differential equations. Math Biosci Eng. 2019;16(5):5419–5450. doi:10.3934/mbe.2019270.

[7] Dingli D, Chalub FACC, Santos FC, Van Segbroeck S, Pacheco JM. Cancer phenotype as the outcome of an evolutionary game between normal and malignant cells. Br J Cancer. 2009;101(7):1130–1136. doi:10.1038/sj.bjc.6605288.

[8] Sato S, Rancourt A, Sato Y, Satoh MS. Single-cell lineage tracking analysis reveals that an established cell line comprises putative cancer stem cells and their heterogeneous progeny. Sci Rep. 2016;6(1):23328. doi:10.1038/srep23328.

[9] Toda M, Martuza RL, Rabkin SD. Tumor Growth Inhibition by Intratumoral Inoculation of Defective Herpes Simplex Virus Vectors Expressing GranulocyteÔÇôMacrophage Colony-Stimulating Factor. Mol Ther. 2000;2(4):324–329. doi:10.1006/mthe.2000.0130.

[10] Randazzo BP, Bhat MG, Kesari S, Fraser NW, Brown SM. Treatment of experimental subcutaneous human melanoma with a replication-restricted herpes simplex virus mutant. J Invest Dermatol. 1997;108(6):933–937. doi:10.1111/1523-1747.ep12295238.

[11] Lee J, Kim Y, Lim J, Kim M, Han K. G-CSF and GM-CSF concentrations and receptor expression in peripheral blood leukemic cells from patients with chronic myelogenous leukemia. Ann Clin Lab Sci. 2008;38(4):331–337.

[12] Liu H. Preclinical evaluation of herpes simplex virus armed with granulocyte-macrophage colony-stimulating factor in pancreatic carcinoma. World J Gastroenterol. 2013;19(31):5138. doi:10.3748/wjg.v19.i31.5138.

[13] Barish S, Ochs MF, Sontag ED, Gevertz JL. Evaluating optimal therapy robustness by virtual expansion of a sample population, with a case study in cancer immunotherapy. Proc Natl Acad Sci. 2017;114(31):E6277–E6286. doi:10.1073/pnas.1703355114.

[14] Carlson JA. Tumor doubling time of cutaneous melanoma and its metastasis. Am J Dermatopathol. 2003;25(4):291–299. doi:10.1097/00000372-200308000-00003.

[15] Meier F, Will S, Ellwanger U, Schlagenhauff B, Schittek B, Rassner G, et al. Metastatic pathways and time courses in the orderly progression of cutaneous melanoma. Br J Dermatol. 2002;147(1):62–70. doi:10.1046/j.1365-2133.2002.04867.x.
