# Supplementary figures and images for: Determinants of combination GM-CSF immunotherapy and oncolytic virotherapy success identified through in silico treatment personalization
Source: PLoS Comput Biol. 2019 Nov 27;15(11):e1007495. doi: 10.1371/journal.pcbi.1007495 (PMC6880985; doi:10.1371/journal.pcbi.1007495)

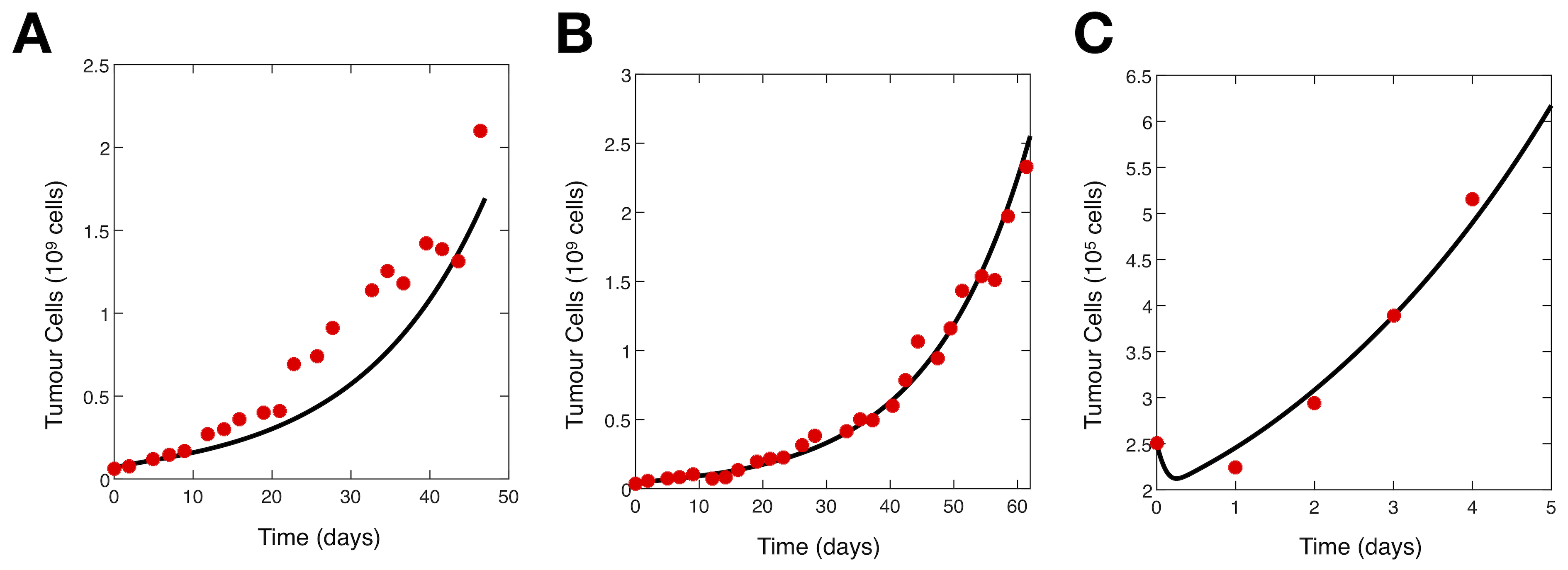

Supplement: S1 Fig — A and B) Data (red circles) from Dingli et al. [31] for tumour growth in immunocompromised mice compared to model predictions (solid black lines). C) Comparisons of model predictions (solid black lines) and the Toda et al. data [32] (red circles) for the number of viable cells following the administration of T-VEC. (TIFF) [file pcbi.1007495.s002.tiff]

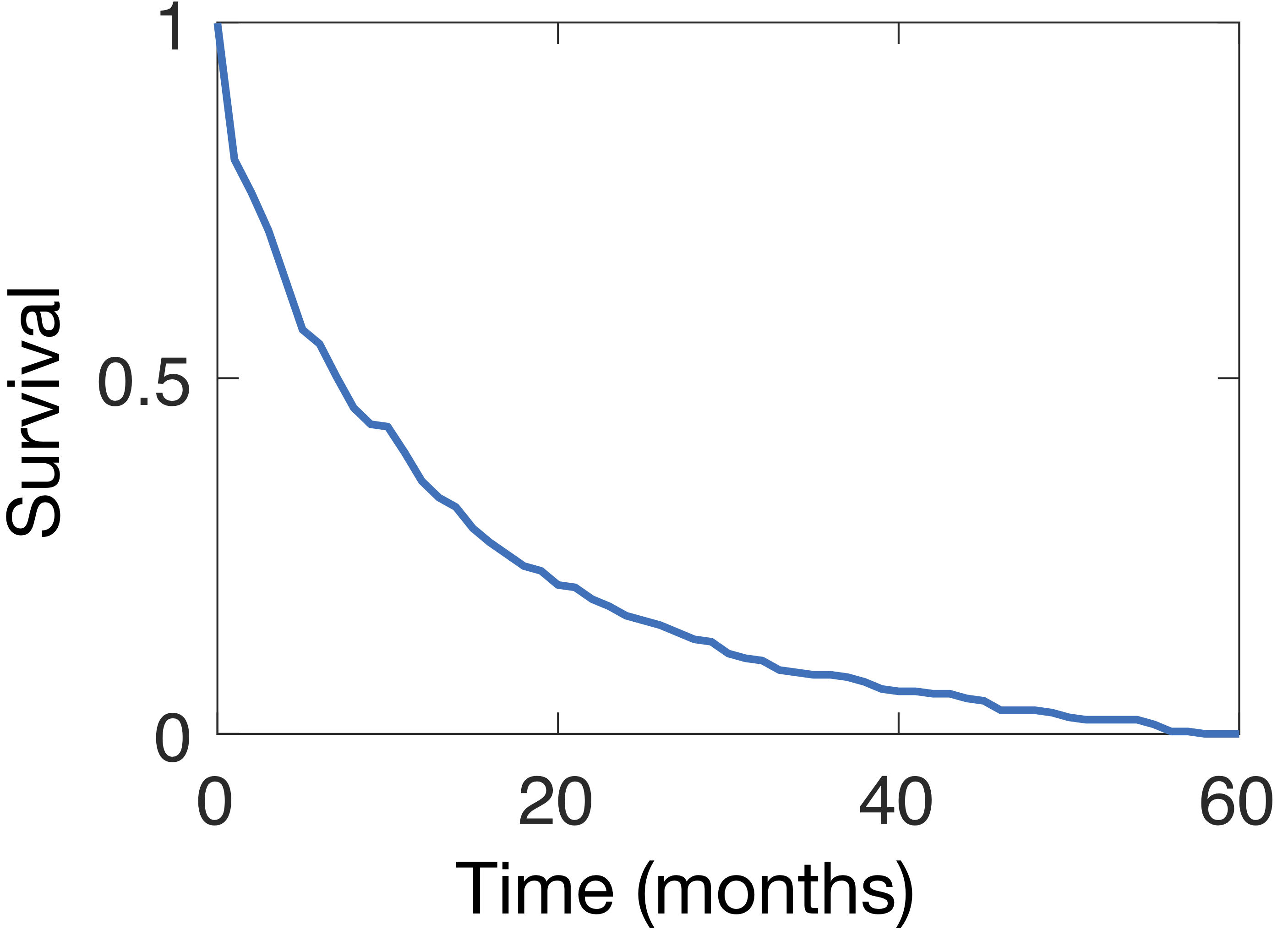

Supplement: S2 Fig — No untreated individual survives to end of 60 month trial. (TIFF) [file pcbi.1007495.s003.tiff]

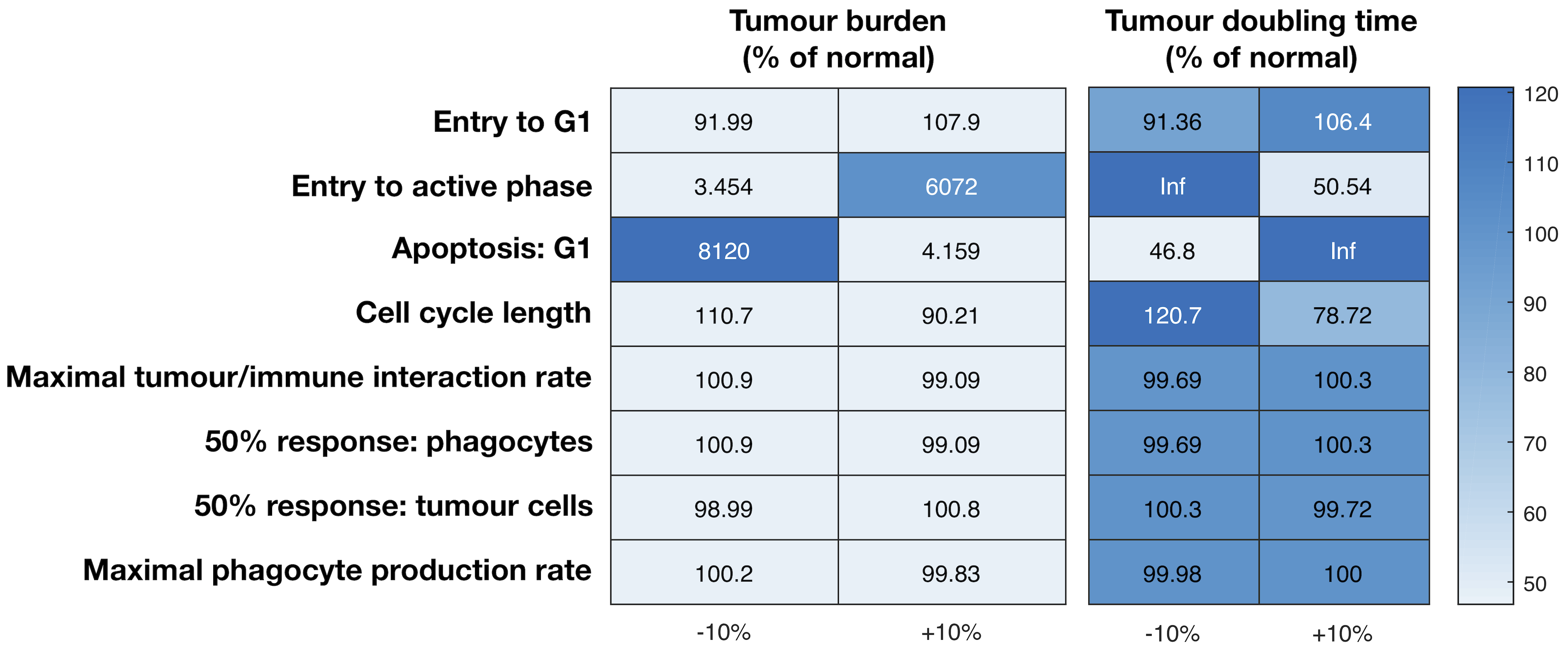

Supplement: S3 Fig — Left: dependence of tumour burden on the parameters shown on the y-axis. Right: dependence of tumour doubling time on the parameters shown on the y-axis. In both cases, parameters were varied by ±10%. Tumour doubling times of Inf indicate that the tumour did not reach twice the initial size. (TIFF) [file pcbi.1007495.s004.tiff]

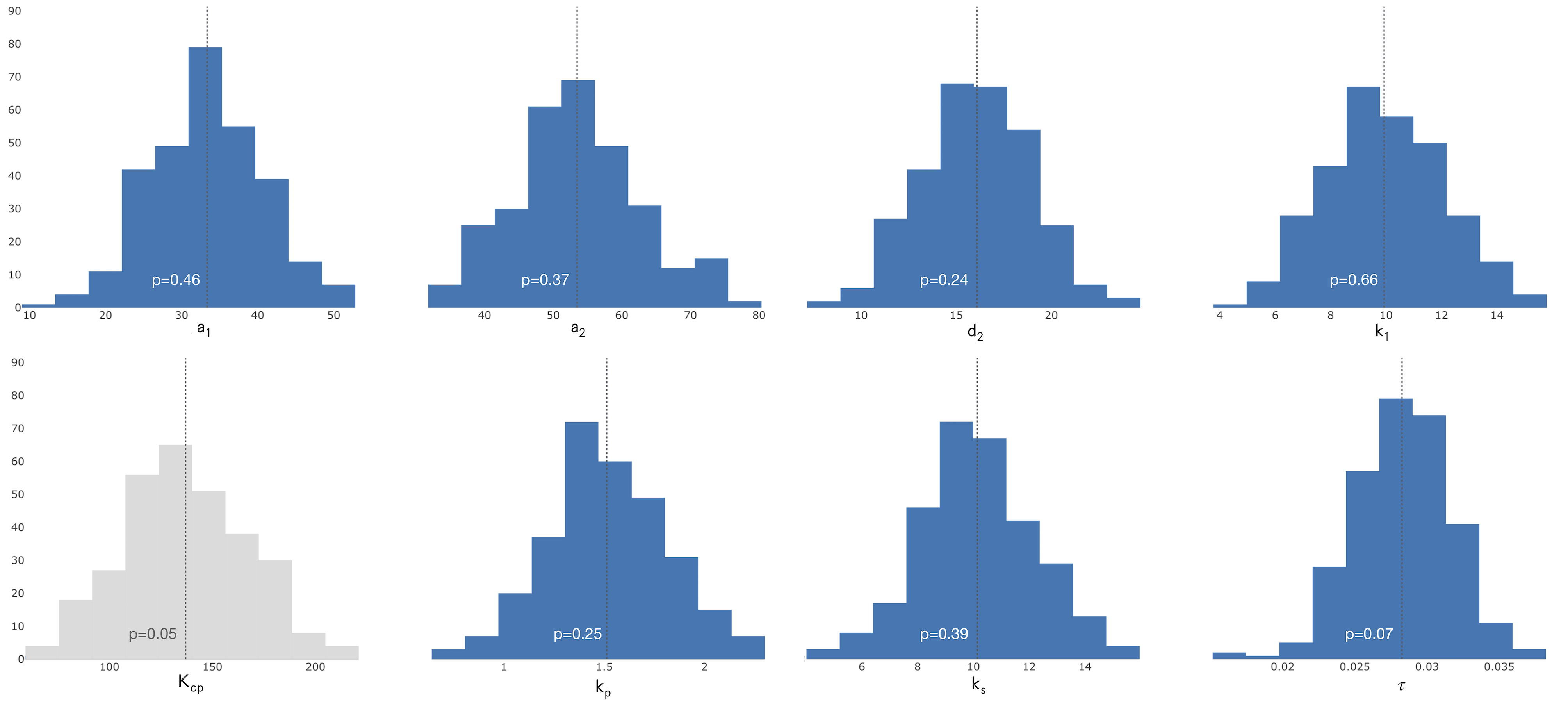

Supplement: S4 Fig — The distributions of the computational biology’s parameters for the 300 in silico individuals in the optimization trial were confirmed to be normal by the Shapiro-Wilk test. Dark blue: determined to be normal at α = 0.05 significance level; grey: weakly normal at α = 0.05 significance level. p-values (indicated on each graph) greater than 0.05 imply no statistically significant difference between parameter distribution and the normal distribution. (TIFF) [file pcbi.1007495.s005.tiff]
