# Supplementary material for: Determinants of combination GM-CSF immunotherapy and oncolytic virotherapy success identified through in silico treatment personalization
Source: PLoS Comput Biol. 2019 Nov 27;15(11):e1007495. doi: 10.1371/journal.pcbi.1007495 (PMC6880985; doi:10.1371/journal.pcbi.1007495)
Supplement: S1 Table — The vector p (see main section Generation of in-silico individuals and patient cohorts) with biological interpretations. See Cassidy and Humphries [28] for detailed descriptions of each parameter. (PDF) [file pcbi.1007495.s006.pdf]

Parameter	Value	Biological Interpretation (Unit)
a_1	1.183	Quiescent to interphase rate (1/day)
d_1	0	Quiescent death rate (1/day)
a_2	1.758	Interphase to active phase rate (1/day)
d_2	0.539	Interphase death rate (1/day)
d_3	0.539	Active phase death rate (1/day)
$\hat{d}_g$	0.167	Active phase death rate (1/day)
κ	3.53	Virion contact rate (1/day)
$\eta_{1/2}$	0.51	Virion half effect concentration (virions)
δ	4.96	Lysis rate (1/day)
α	0.00829	Lytic virion release rate (virions/cell)
ω	9.686	Virion death rate (1/day)
k_{cp}	4.675	Maximal phagocyte production rate (10^{10} cells/day)
$C_{1/2}$	0.739	Phagocyte production half effect (ng/mL/day)
$\Psi_{1/2}$	5	Cytokine production half effect (10^{10} cells/day)
γ_p	0.35	Phagocyte death rate (1/day)
C^*_{prod}	3.98×10^{-4}	Homeostatic cytokine production rate (ng/mL/day)
C^{max}_{prod}	1.429	Maximal cytokine production rate (ng/mL/day)
k_{elim}	0.16139	Cytokine elimination rate (1/day)
k_p	0.05	Phagocyte-tumour cell contact rate (1/day)
$k_{q,s}$	10	Phagocyte cell digestion constant
τ	0.8354	Expected cell cycle duration (day)
